# Increased Rank for Medical Officers

**Published:** 1918-10

**Authors:** 


### INCREASED RANK FOR MEDICAL OFFICERS

The Owen Bill, which provided that medical officers in the United States Army be given the same relative rank as those of the Navy, and that the proportion of general officers in the Medical Department be the same as in the other branches of the service, has been defeated. However, a substitute bill was passed as an amendment, or "rider" to the Army appropriation bill, which provides for one major-general and two brigadier-generals in the Medical Reserve Corps. This amendment provides for an increase in the grades from colonel down, which will be relatively greater than the number of general officers; but it is not known yet how much this will affect the medical officers of the American Expeditionary Forces.

A recent order from the President obliterates the lines of distinction between the Regular Army, the National Guard, the National Army and the Reserve Corps, and brings every officer and soldier serving under the American flag into the United States Army for the period of the war. Whether or not this order will affect the administration of this act is not understood by the writer, but it is certain that the Chief Surgeon will secure as many promotions as he is allowed to do for the medical officers serving in France and England.

This legislation, though it does not adequately recognize the Regular Medical Corps, or the Medical Reserve Corps, will help to give the Medical Department of the Army the standing which it deserves; and judging from the laudatory remarks of Senators regarding the work and sacrifices of the members of the medical profession in serving their country in this crisis, there is hope that during the next Congress, which assembles in December, more liberal provision will be made for both the regular and reserve medical officers of the army.

### The Amended Bill.

The following is the text of the Amendment 65 to the Army Appropriation Bill providing for an increase in the Medical Department:

"The Medical Department of the Regular Army is hereby increased by one assistant surgeon-general, for service abroad during the present war, who shall have the rank of major-general, and two assistant surgeons-general, who shall have the rank of a brigadier-general, all of whom shall be appointed from the Medical Corps of the Regular Army."

"The President may nominate and appoint in the Medical Department of the National Army, by and with the advice and consent of the Senate, from the Medical Reserve Corps of the Regular Army not to exceed two major generals and four brigadier generals."

"The commissioned officers of the Medical Corps of the Regular Army, none of whom shall have rank above that of colonel shall be proportionately distributed in the several grades as now provided by law."

"The commissioned officers of the Medical Reserve Corps of the Regular Army, none of whom shall have rank above that of colonel, shall be proportionately distributed in the several grades, as now provided by law for the Medical Corps of the Regular Army, provided that nothing in this act shall be held or construed so as to discharge any officer of the Regular Army or deprive him of a commission which he now holds therein."

